# Estimation of Soil-Water Characteristic Curves in Multiple-Cycles Using Membrane and TDR System

**DOI:** 10.3390/ma9121019

**Published:** 2016-12-17

**Authors:** Won-Taek Hong, Young-Seok Jung, Seonghun Kang, Jong-Sub Lee

**Affiliations:** School of Civil, Environmental and Architectural Engineering, Korea University, 145 Anam-ro, Seongbuk-gu, Seoul 136-713, Korea; 01698788767@korea.ac.kr (W.-T.H.); mcaie@korea.ac.kr (Y.-S.J.); gshnice@korea.ac.kr (S.K.)

**Keywords:** membrane, soil-water characteristic curve, time domain reflectometry, unsaturated soils, volumetric pressure plate extractor

## Abstract

The objective of this study is to estimate multiple-cycles of the soil-water characteristic curve (SWCC) using an innovative volumetric pressure plate extractor (VPPE), which is incorporated with a membrane and time domain reflectometry (TDR). The pressure cell includes the membrane to reduce the experimental time and the TDR probe to automatically estimate the volumetric water content. For the estimation of SWCC using the VPPE system, four specimens with different grain size and void ratio are prepared. The volumetric water contents of the specimens according to the matric suction are measured by the burette system and are estimated in the TDR system during five cycles of SWCC tests. The volumetric water contents estimated by the TDR system are almost identical to those determined by the burette system. The experimental time significantly decreases with the new VPPE. The hysteresis in the SWCC is largest in the first cycle and is nearly identical after 1.5 cycles. As the initial void ratio decreases, the air entry value increases. This study suggests that the new VPPE may effectively estimate multiple-cycles of the SWCC of unsaturated soils.

## 1. Introduction

Most soil near the surface consists of an unsaturated zone with soil particles, water, and air. The essential component of unsaturated soils is the matric suction. The matric suction in unsaturated soils is sensitive to the environmental conditions [1,2]. For example, the matric suction of unsaturated soil increases in drying conditions, while the matric suction decreases in wetting conditions. Matric suction changes alter the characteristics and mechanical behavior of unsaturated soils. For the prediction of the shear strength of the unsaturated soils, Vanapalli et al. [3] developed an analytical model using the soil-water characteristic curve (SWCC) and the effective shear strength parameters. In addition, the matric suction of the unsaturated soils significantly affects the resilient modulus and the permanent deformation of the ground [4,5]. Therefore, understanding matric suction is important to analyze the properties of unsaturated soils. Matric suction can be estimated by the soil-water characteristic curve, which is the relationship between matric suction and the volumetric water content or the degree of saturation in the soil [6].

The SWCC is dependent upon on the soil type, grain size distribution, initial void ratio, and plasticity. The SWCC generally shows hysteresis behavior: the volumetric water content of the drying process is higher than that of the wetting process with the same matric suction. Most of the SWCC, however, is generally obtained for the drying process for the convenience of the measurement [2]. A ceramic disk with a high air entry value has been commonly used to obtain SWCCs; therefore, it takes an extremely long time to obtain a SWCC in the wetting process because of the difficulty of equilibrium conditions [7]. In this study, a microporous membrane was used to reduce the experimental time instead of a high air entry ceramic disk. The microporous membrane has high hydraulic conductivity and is thin, so the experimental time was significantly reduced. Thus, this new apparatus incorporated with a membrane could conduct multiple-cycles of SWCC tests within a short experimental time.

Quantitative measurements of the volumetric water content of unsaturated soils have been commonly conducted using the burette system during the SWCC tests. The burette system provides a continuous measurement of the soil water content, which is recommended to minimize errors in the identification of the SWCC [8]. In this study, the volumetric water contents of unsaturated soils were estimated by the time domain reflectometry (TDR) system as well as the burette system. The TDR can be used to monitor continuous changes in the soil water content [9]. Furthermore, the TDR probe calibrations for the J30-50 (Jumunjin sand) and F100 (Fine sand) sands were performed to accurately estimate the soil water content. The volumetric water content estimated by the TDR system during the SWCC tests is compared with that measured by the burette system, and the result exhibits a direct proportional relationship. Thus, the TDR system could effectively be used for the measurement of water content during the SWCC tests.

In this study, SWCC tests with different initial void ratios and particle sizes were performed to characterize the properties of the SWCC. First, background on the fundamental components of unsaturated soils such as soil suction, the soil-water characteristic curve and the newly developed time domain reflectometry system are explained. Second, the features of the experimental setup and experimental studies are described. Third, the experimental results according to the filter type and particle size are compared. Subsequently, the property changes of SWCC according to multiple-cycles, initial void ratio, and particle size are presented. In addition, the results of the TDR measurements were compared with those of the burette system. Lastly, the results of multiple-cycles of the SWCC test are discussed.

## 2. Characterization of Unsaturated Soils

### 2.1. Soil Suction

The soil suction (ψ) consists of a matric suction (*u*_a_ − *u*_w_) and osmotic suction (π), as follows [1]

ψ = (*u*_a_ − *u*_w_) + π (1)

where the matric suction (*u*_a_ − *u*_w_), which indicates the difference between air pressure (*u*_a_) and pore-water pressure (*u*_w_), is the net pressure applied to soil particle–air interface. Note that matric suction has a same absolute value to the matric potential in a stress state variable [10] with opposite sign. The matric suction changes depending on climatic conditions, such as drying and wetting conditions. As the radius of curvature of the meniscus decreases in the drying process, the matric suction increases. Conversely in the wetting process, the matric suction decreases. Thus, the variation of matric suction causes alternation in the unsaturated soil properties. The osmotic suction (π), which is the tendency of a solution to take in water, varies according to the chemical component, the concentration of ions, and the fraction of the soil particles. The osmotic suction causes water to move on the high concentration side.

### 2.2. Soil-Water Characteristic Curve

**Figure 1:**
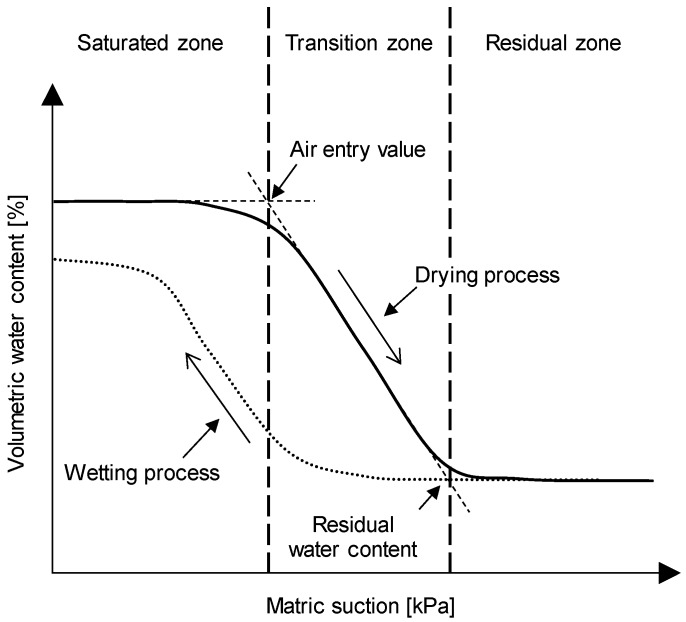
Typical soil-water characteristic curve.

The soil-water characteristic curve (SWCC) represents the relationship between matric suction and the volumetric water content [11]. The SWCC is dependent upon the mineralogy, particle size distribution, soil type, dry unit weight, and permeability of the unsaturated soil, so the SWCC has been used to estimate the shear strength and tensile strength of the unsaturated soils. Furthermore, the flow of groundwater and the slope stability of unsaturated soils can be investigated with the SWCC. A typical soil-water characteristic curve is plotted in Figure 1, which shows that the SWCC can be categorized into saturated, transition, and residual zones [3]. In the saturated zone, pore-water is not flowing out until the matric suction is greater than the air entry value (AEV), which is the intersection of the extensions of the tangent lines in the saturated zone and the transition zone, as shown in Figure 1. In the transition zone, the air is flowing into the pore as the matric suction increases and thus pore-water flows out. The characteristics of the transition zone determine the slope of the SWCC. For the residual zone, while the matric suction increases, no water is flowing out. The constant volumetric water content, which is called the residual water content, is maintained.

### 2.3. Time Domain Reflectometry

**Figure 2:**
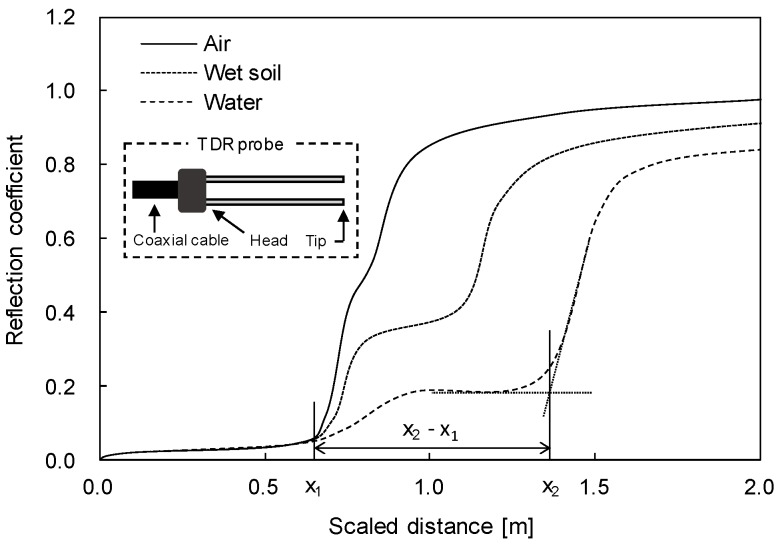
Typical TDR waveforms in air, wet soil, and water.

The time domain reflectometry (TDR) system consists of a pulse generator, a coaxial cable and a TDR probe. Note that the TDR probe consists of two or three slender rods [12]. In this study, a two—rod type probe is used in order to minimize the effect of the soil disturbance during the wetting and drying processes [13]. In the TDR system, the propagation time of the guided electromagnetic wave is measured for the estimation of the dielectric constant of soils around the TDR probe. The electromagnetic wave, which is generated by the pulse generator, propagates through the coaxial cable and the TDR probe. The electromagnetic wave reflected at the head and the tip of the TDR probe (see Figure 2), due to the impedance mismatch, is collected in time domain.

The Typical TDR waveforms in air, wet soil, and water are represented in Figure 2. The waveform of the reflected electromagnetic wave is the function of the probe configuration and the soil properties around the TDR probe. As the probe configuration is fixed, the shape of the reflected wave is mainly dependent upon the dielectric constant of the surrounding soils. The reflection time at the probe tip increases as the dielectric constant increases and the scaled distance increases in the TDR waveform. From the scaled distance (horizontal axis in Figure 2), the apparent length (*x*_2_ − *x*_1_) can be calculated and the dielectric constant can be evaluated as follows [12]

κ = [(*x*_2_ − *x*_1_)/*L*]^2^ (2)

where κ is the dielectric constant; *x*_1_ and *x*_2_ are reflection points at the beginning (interface between the coaxial cable and the TDR probe) and the tip of the TDR probe; and *L* is the TDR probe length.

The dielectric constant of the unsaturated soils varies sensitively depending on the volumetric water content. Thus, the volumetric water content that is required in the SWCC can be estimated by using the TDR system. The most commonly used relationship between the dielectric constant (κ) and volumetric water content (θ_v_) is as follows [14]

θ_v_ = *a*κ^3^ + *b*κ^2^ + *c*κ + *d* (3)

where the coefficients of *a*, *b*, *c*, and *d* are experimentally determined. Topp et al. [14] suggested *a* = 4 × 10^−6^, *b* = −5.5 × 10^−4^, *c* = 2.92 × 10^−2^, and *d* = −5.3 × 10^−2^. If the error of the volumetric water content is approximately 0.02–0.03 m^3^·m^−3^, the coefficients of *a*, *b*, *c*, and *d* should be determined in the calibration phase [15].

## 3. Experimental Setup and Studies

### 3.1. Specimens

**Figure 3:**
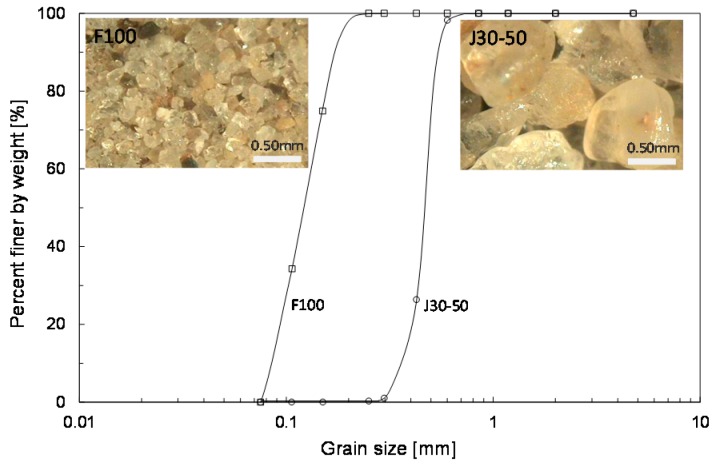
Particle size distribution of J30-50 and F100 sands.

The experimental studies were conducted using J30-50 and F100 sands. The grain-size distributions of the two specimens are plotted in Figure 3. Figure 3 shows that both J30-50 and F100 are uniform specimens. For the J30-50 sand, the sand passes the sieve No. 30 and remains on sieve No. 50. The F100 sand is a sample with a grain size between sieve No. 100 and sieve No. 200. The index properties of both sands are summarized in Table 1. The mean diameters (D_50_) are 0.46 mm and 0.13 mm for the J30-50 and F100 sands, respectively. The specific gravities [16] are 2.62 and 2.65 for the J30-50 and F100 sands, respectively. The maximum void ratio [17] and the minimum void ratio [18] are 0.99 and 0.62 for the J30-50 sand, respectively. For the F100 sand, the maximum void ratio and minimum ratio are 0.96 and 0.59, respectively. According to the unified soil classification system (USCS), both J30-50 and F100 sands are classified as poorly graded sandy soils (SP).

### 3.2. Volumetric Pressure Plate Extractor

For the measurement of the SWCC in unsaturated soils, several methods such as a methylene blue test [19], a tempe pressure cell, a thermal conductivity cell [20,21], thermocouple psychrometers [22] and a pressure plate apparatus have been used. In addition, several analytical and mathematical models using grain size distribution and the other properties of the unsaturated soils were established for the prediction of the SWCC [23]. However, the methods related with the thermal characteristics of the unsaturated soils indirectly measure the SWCC, and the analytical and mathematical methods require the experimental backgrounds. Among the SWCC test methods, the pressure plate apparatus, which utilizes the axis-translation technique, is most commonly used because the pressure plate apparatus allows the direct measurement of the water content under the applied matric suction acted on a specimen. The pressure plate apparatus is divided into two types, depending on the measurement method. First, the pressure plate extractor (PPE) type measures the weight change of the sample. Second, the volumetric pressure plate extractor (VPPE) evaluates the volume of water flowing out from the sample. Note that the PPE type may not generate the wetting curve of the SWCC and may disturb the sample during weight measurements of the specimens. Thus, the VPPE has been commonly used to continuously obtain the drying and wetting processes of the SWCC.

**Figure 4:**
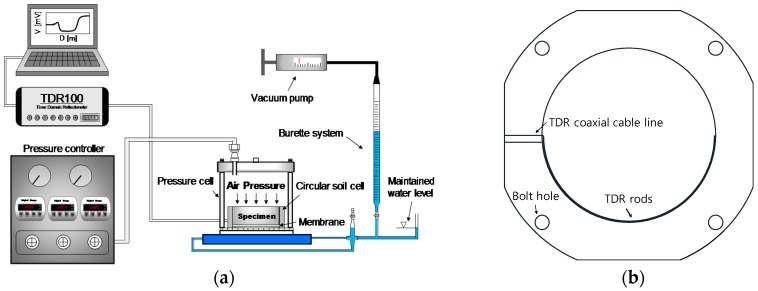
Schematic drawing of apparatus: (**a**) VPPE incorporated with the TDR system; (**b**) circular soil cell.

In this study, the VPPE is used to determine the SWCC. The VPPE may change the soil water content by adjusting the matric suction. The VPPE system mainly consists of a pressure controller, a burette system, and a cylindrical pressure cell as shown in Figure 4a. As shown in Figure 4a, the low-pressure regulator (0–70 kPa) is used as a pressure controller and was adopted for the evaluation of the drying and the wetting processes. Additionally, a digital gauge was installed to accurately determine the air pressure applied in the specimens. The inflow and outflow volumes of the pore water in the drying and wetting processes were measured by the burette system and estimated by the TDR system as shown in Figure 4a.

A membrane, instead of a high air entry ceramic disk, was placed between the soil specimen and the porous stone to reduce the equilibrium time of the matric suction due to the thin (140 μm) membrane thickness and the high hydraulic conductivity in the membrane [7]. The high air entry ceramic disks, which have been commonly used in the typical SWCC tests [24], require a significantly longer equilibrium time in matric suction [25,26,27]. The specifications of a typical ceramic disk and membrane are compared in Table 2.

Note that the values of hydraulic conductivity are about 10^−5^–10^−8^ m/s and ~10^−8^ m/s for the silt and the clay, respectively. Therefore, the experimental time of the SWCC test incorporated by the membrane can be significantly reduced even for the fine soils.

The circular soil cell shown in Figure 4b was manufactured using polyamide material to minimize the effects of interference in the electromagnetic waves during the TDR tests. The dimensions of the circular soil cell are 73 mm in inner diameter and 30 mm in height.

### 3.3. Time Domain Reflectometry System

The time domain reflectometry (TDR) system consists of TDR electronics, a coaxial cable, and a TDR probe. The coaxial cable is a RG 58 C/U type and the cable length is determined as 3 m, which is the minimal length for the measurement system. As the cable length becomes longer, more uncertainty in the determination of reflection time is introduced [28]. The schematic drawing of the TDR probe installed in the circular soil cell is shown in Figure 4b. The TDR probe is made of stainless steel and is a curved two-rod type. The diameter and length of the rods used in the TDR probe are 3 mm and 110 mm, respectively. As the curvature of the two rods is identical with that of the circular cell, the two-rod type TDR probe was embedded horizontally into the inside of the circular soil cell as shown in Figure 4b. At each stage of the electromagnetic wave measurement, 256 signals were averaged to minimize the uncorrelated noises.

### 3.4. TDR Probe Calibration

**Figure 5:**
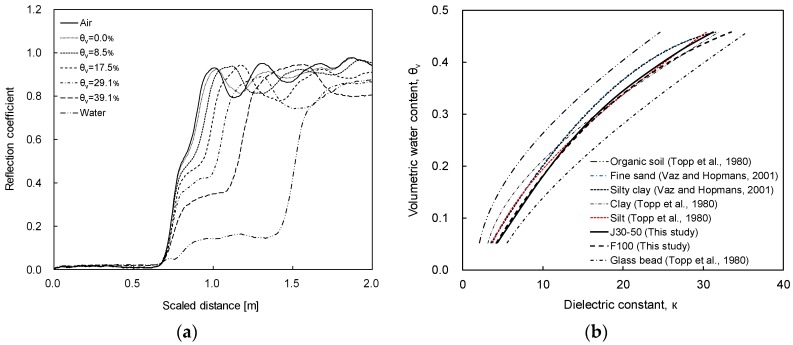
TDR calibration: (**a**) TDR waveform for J30-50 sand; (**b**) relationship between the dielectric constant and the volumetric water content. θ_v_ denotes the volumetric water content.

Probe calibration is essential for the accurate estimation of the volumetric water contents of the soil specimens. For the derivation of the accurate relationship between the volumetric water content and the dielectric constant, the calibration in water and air should be included [29,30]. Thus, the calibration of the TDR probe was conducted in water, air, and J30-50 and F100 sands at the different volumetric water contents. For TDR probe calibrations and SWCC tests, distilled water is used to control the volumetric water content of the specimens. The waveforms captured in the air, water, and J30-50 sands with a wide range of volumetric water contents are plotted in Figure 5a. Figure 5a shows that the scaled distance increases with an increase in the volumetric water content of soils. The evaluated dielectric constant was plotted corresponding to the volumetric water content for both J30-50 and F100 sands, as shown in Figure 5b. In addition, the relationships between the dielectric constant and the volumetric water content of other soils reported in other researches [14,31] are overlaid in Figure 5b. Figure 5b shows that J30-50 and F100 sands are placed in the middle of other soils. The relationship between the dielectric constant and the volumetric water contents for J30-50 and F100 sands are

J30-50 sand: θ_v_ = 4 × 10^−6^κ^3^ − 5.5 × 10^−4^κ^2^ + 3.02 × 10^−2^κ − 7.1 × 10^−2^ (4)

F100 sand: θ_v_ = 4 × 10^−6^κ^3^ − 5.5 × 10^−4^κ^2^ + 2.92 × 10^−2^κ − 6.3 × 10^−2^ (5)

### 3.5. Test Procedure

The experimental studies for the estimation of the SWCCs are conducted as follows. First, the porous stone is installed on the base of the VPPE system and fully saturated. Subsequently, the membrane is placed above the porous stone and is fully saturated to minimize the influence of air. After the saturation is completed, a circular soil cell with an O-ring is installed on the porous stone and the membrane. A bolt and a nut are used in the circular soil cell to turn the airflow off in the VPPE system. The specimen with the specified initial void ratio is prepared by mixing the sand and water. After the sand specimen is placed into the circular soil cell, water is filled from the outside of the specimen and the soil specimen is soaked for at least 24 h for full saturation. When the setting of the soil specimen is completed, the pressure cell is connected to the pressurized air supply, and air pressure is gradually applied using the pressure controller. The range of the applied matric suction is 0.1–60.0 kPa. For the identification of the SWCC of unsaturated soils, the matric suction should be applied up to 50 kPa in the drying process and up to 0.1 kPa in the wetting process [8]. Five cycles of the drying and wetting processes are completed to investigate the variation of the SWCC according to the repeated drying–wetting processes. Simultaneously, when the water level in the burette is stabilized under the applied matric suction, the inflow–outflow volume of the pore water is measured using the burette system to determine the volumetric water content. Additionally, the electromagnetic waves of the TDR are measured at each matric suction to estimate the volumetric water content.

## 4. Experimental Results and Analyses

As experimental studies, several cases of SWCC tests are conducted for the specimens with the different initial void ratio and particle size for the estimation of the SWCCs of different types of unsaturated specimens. The volumetric water contents estimated by the TDR system are compared with those determined by the burette system. Furthermore, the experimental time, matric suction, and volumetric water content are continuously monitored during the SWCC tests.

### 4.1. Water Content

**Figure 6:**
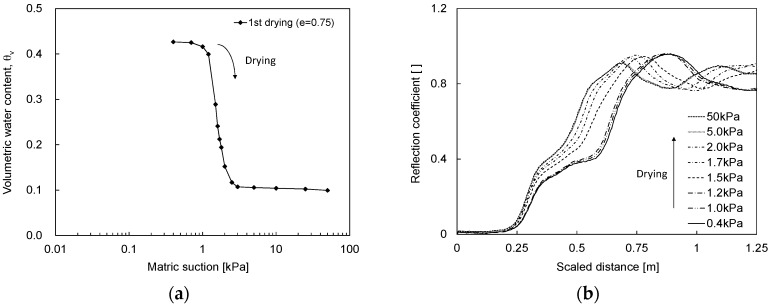
SWCC and TDR waveform during the drying process: (**a**) SWCC; (**b**) TDR waveform.

The TDR waveforms according to the matric suction and corresponding SWCC are investigated for J30-50 sand with an initial void ratio of 0.75. In the drying process, as the matric suction increases, the volumetric water content measured by the burette system decreases from 0.425 to 0.1 as shown in Figure 6a and the TDR waveform shifts from the right side to the left side as shown in Figure 6b. Thus, the apparent length in the TDR signature decreases, and the dielectric constant decreases. In the drying process, the significant change of the apparent length of the waveform occurs in the matric suction of 1.2–1.5 kPa, which corresponds to the AEV.

**Figure 7:**
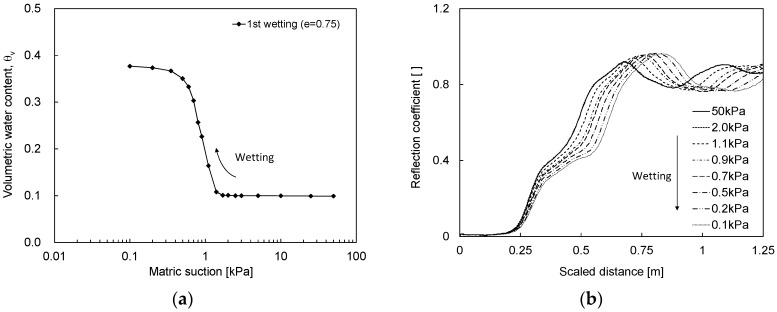
SWCC and TDR waveform during the wetting process: (**a**) SWCC; (**b**) TDR waveform.

In the wetting process, as the matric suction decreases, the volumetric water content measured by the burette system increases from 0.1 to 0.375 as shown in Figure 7a. In addition, the opposite phenomenon occurs in the TDR waveforms as shown in Figure 7b. As the matric suction decreases from 50 kPa, the waveforms in the matric suction of 50–2 kPa are nearly identical.

**Figure 8:**
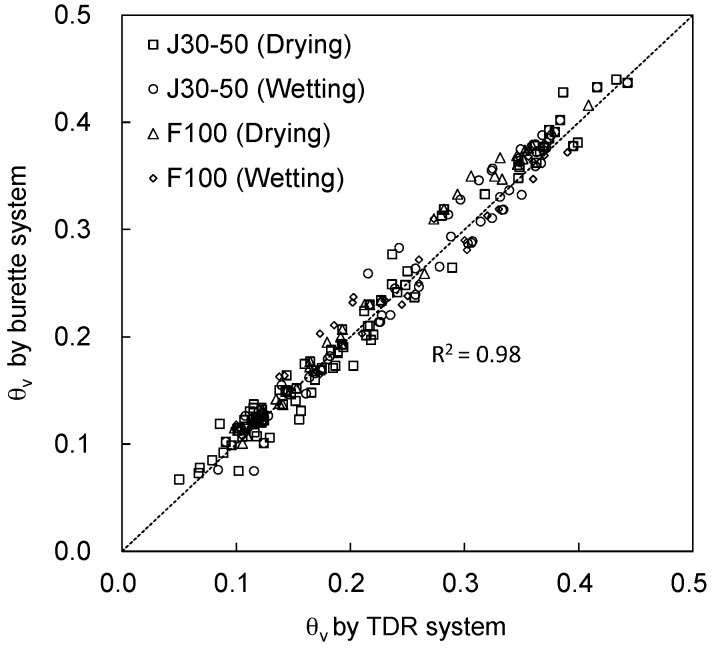
Volumetric water content comparison.

The dielectric constant during the drying and wetting phases were evaluated from the TDR waveforms. The volumetric water content was estimated by substituting the dielectric constant into the calibration results as expressed in Equations (4) and (5). Then, the volumetric water contents estimated by the TDR system are compared with those determined by the burette system for J30-50 and F100 sands as shown in Figure 8. Figure 8 shows that the volumetric water contents evaluated by the TDR and the burette systems are very similar for both sand specimens. The determinant coefficient (*R*^2^) is 0.98 for J30-50 and F100 sands. The R^2^ is significantly high, so the volumetric water content evaluated by the TDR system can be effectively used, if the automatic TDR system is prepared.

### 4.2. Experimental Time

**Figure 9:**
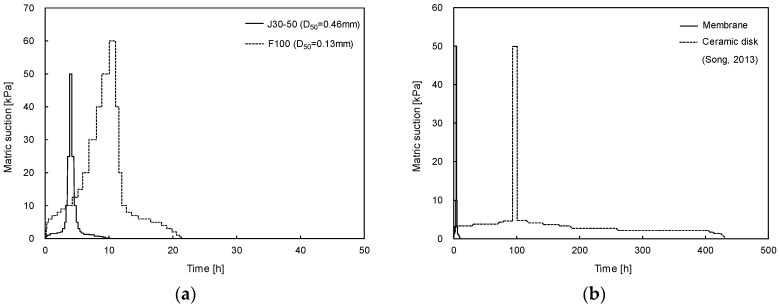
Experimental time for one cycle of the SWCC: (**a**) membrane for J30-50 and F100 sands with an initial void ratio of 0.85; (**b**) membrane and ceramic disk for J30-50 sand with an initial void ratio of 0.75.

The experimental time for one cycle of the SWCC for the J30-50 and F100 sands with an initial void ratio of 0.85 is plotted in Figure 9a, which is obtained with use of the membrane. For F100 sand, the experimental time of one cycle of the drying and wetting processes is approximately 22 h. The experimental time for J30-50 sand (~10 h) is shorter than that of F100 sand because of the larger particle and pore size of J30-50 sand. For comparison, the experimental time with the ceramic disk for the J30-50 sand, with the same initial void ratio of 0.75, is plotted in Figure 9b. While the ceramic disk has been commonly used for the determination of the SWCC, the experimental time is considerably long [32]. For example, for J30-50 sand with an initial void ratio of 0.75, approximately 18 days were required for one cycle of the drying and wetting processes. However, the experimental time with a membrane is less than 10 h for J30-50 sand with an initial void ratio of 0.75. Thus, the experimental time is considerably shorter with membrane use because of the shorter travel distance with higher hydraulic conductivity in the membrane, as summarized in Table 2. The modified volumetric pressure plate using the membrane may significantly reduce the experimental time, so the multiple-cycles of the SWCC tests can be conducted. Note that the most geotechnical problems are associated with the wetting process [33,34]. Most SWCC studies, however, have been commonly performed on the drying process of one cycle due to the experimental time [2].

### 4.3. SWCC

#### 4.3.1. Hysteresis in SWCC

**Figure 10:**
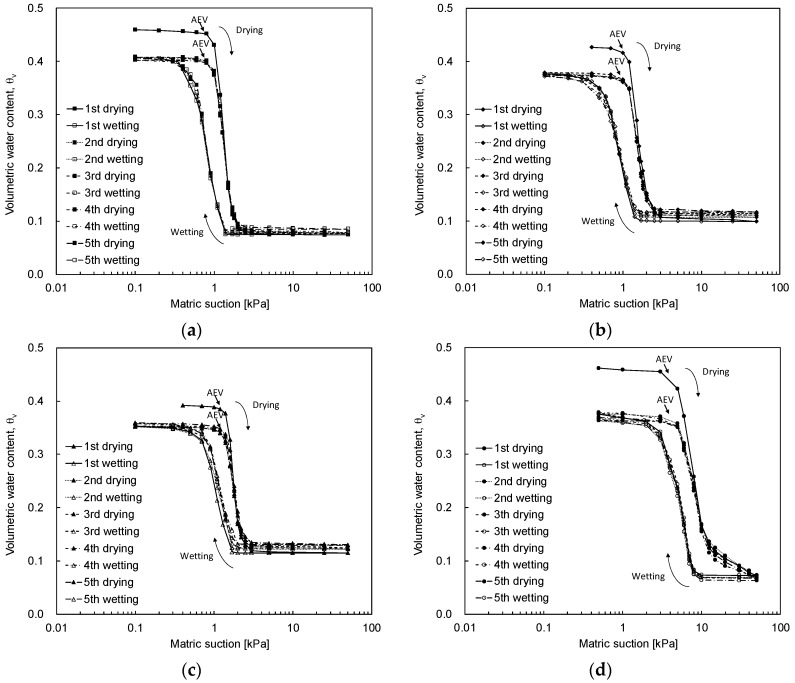
Multiple-cycles of the soil-water characteristic curve: (**a**) void ratio of *e* = 0.85 for J30-50 sand; (**b**) void ratio of *e* = 0.80 for J30-50 sand; (**c**) void ratio of *e* = 0.75 for J30-50 sand; (**d**) void ratio of *e* = 0.85 for F100 sand. AEV denotes the air entry value.

The soil-water characteristic curves obtained with the prepared specimens are plotted in Figure 10. The volumetric water content decreases in the drying process and increases in the wetting process. The drying curve shows the water desorption of the specimen as the matric suction increases. In contrast, the wetting curve shows the water adsorption of the specimen in accordance with the decrease in the matric suction.

All SWCCs show hysteresis behavior: the drying curves have a higher volumetric water content at the same matric suction [1] due to the ink bottle effect, contact angle effect, and soil fabric change during the drying and wetting processes [35,36]. As the pore-size and shape of the unsaturated soils are non-uniform, the radius of curvature and the contact angle between the soil and air-water are not identical during the wetting and drying processes. The radius of curvature and contact angle in the wetting process are higher than those in the drying process. The soil with a higher curvature and higher contact angle has an easier time desorbing the water; therefore, the water content of soil during the wetting process is lower. Figure 10 shows that after 1.5 cycle of the SWCC (first drying–first wetting–second drying), the shape and size of the SWCCs are almost identical. Thus, more than 1.5 cycles of the SWCC tests are required to fully characterize the SWCC behavior of unsaturated soils. Additionally, the hysteresis size of the first cycle for both sands is greater than that of the other cycles, as shown in Figure 10. The hysteresis magnitudes after the second cycle are almost identical.

#### 4.3.2. Initial Void Ratio Effect

**Figure 11:**
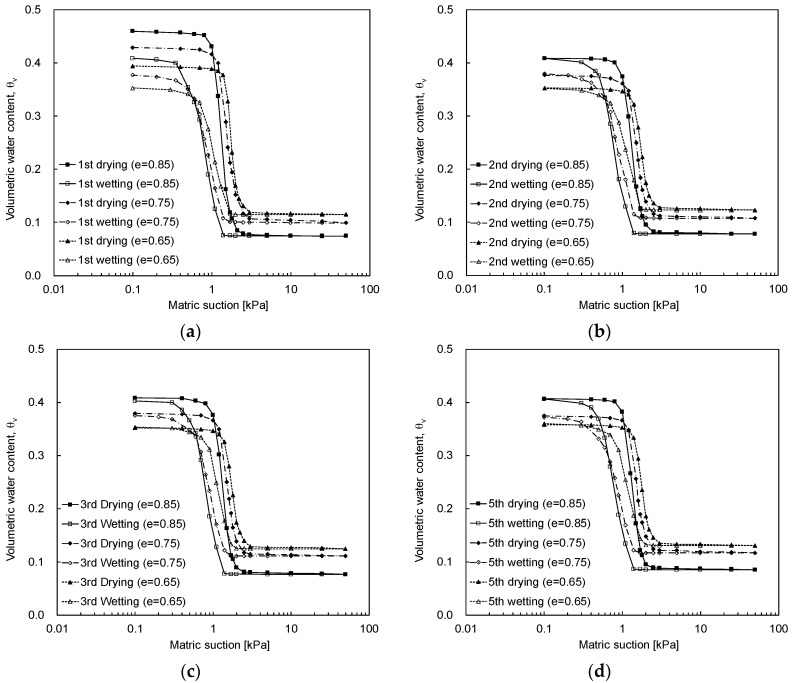
SWCC according to the initial void ratio: (**a**) first cycle; (**b**) second cycle; (**c**) third cycle; (**d**) fifth cycle.

The SWCCs according to the initial void ratio are represented in Figure 11 for J30-50 sand at the first, second, third, and fifth cycles. As the initial void ratio increases in J30-50 sands, the air entry value (AEV) decreases, and the volumetric water content corresponding to the AEV increases as summarized in Table 3.

As the initial void ratio of the specimen decreases, the matric suction for the residual zone (Figure 1) increases because the flow of pore-water decreases in the smaller pore-sized specimens [37,38,39]. Furthermore, the difference in water content from the saturated zone to the residual zone increases with an increase in the initial void ratio as summarized in Table 4. The SWCC trend obtained in the first cycle is similar to that of previous studies [37,38,40]. However, the SWCC trends change as the number of cycles increases. Therefore, multiple-cycles of SWCC are necessary to characterize fully the behavior of the unsaturated soils.

#### 4.3.3. Particle Size Effect

**Figure 12:**
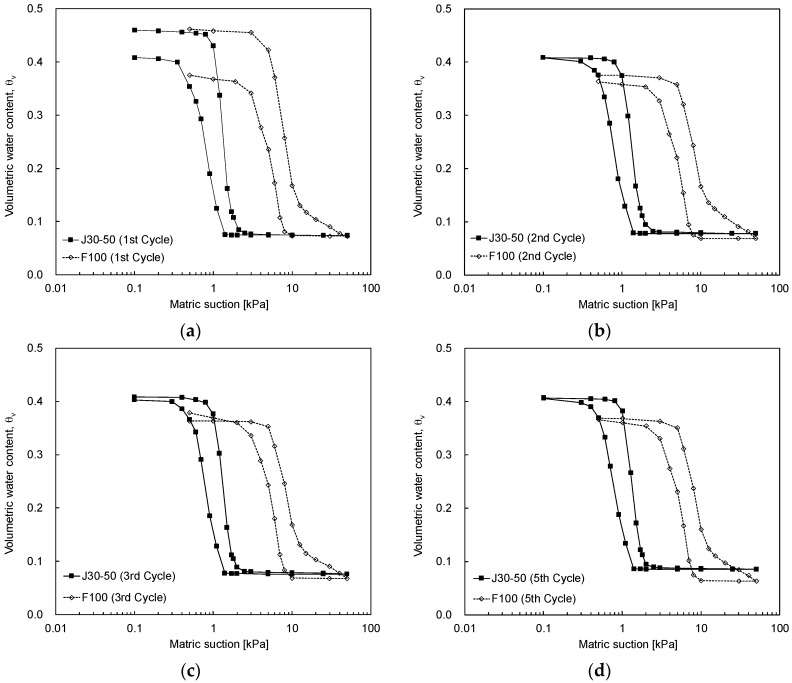
SWCC according to the particle size: (**a**) first cycle; (**b**) second cycle; (**c**) third cycle; (**d**) fifth cycle.

The influence of particle size on the SWCC is observed in Figure 12. The mean diameter of J30-50 sand (0.46 mm) is 3.5 times greater than that of F100 sand (0.13 mm) at the same initial void ratio (0.85). Figure 12 shows that as the fine content decreases, the SWCC shifts to the left side. Similar results can be found in previous studies [27,41,42]. While the initial void ratio for both specimens is identical, the matric suction of F100 sand is greater than that of J30-50 sand with the same volumetric water content. Thus, the AEVs for J30-50 and F100 sands at an initial void ratio of 0.85 are 1.0 kPa and 5.0 kPa, respectively, as summarized in Table 3. In addition, J30-50 sand shows the steeper SWCC relationship. The total hysteresis size, which is the sum of the differences in the volumetric water content between the drying curve and the wetting curve, shows similar results to those found in previous studies [38]. The total hysteresis size is in inverse proportion to the particle size as shown in Figure 12.

## 5. Summary and Conclusions

For the estimation of multiple-cycles of the soil-water characteristic curve (SWCC), the innovative volumetric pressure plate extractor (VPPE), which was incorporated with a membrane and time domain reflectometry (TDR), was introduced. A membrane was used instead of a ceramic disk, and the TDR system was adopted to automatically estimate the volumetric water content of the soil specimen. Note that the experimental time for the membrane VPPE was compared with that of the ceramic disk VPPE, and the volumetric water contents estimated by the TDR system are compared with those measured by the burette system. By using the innovative VPPE, multiple-cycles of SWCCs are obtained for the sand specimens with different initial void ratios and different particle sizes. Jumunjin sand (J30-50) with a mean diameter of 0.46 mm, and fine sand (F100) with a mean diameter of 0.13 mm were used. For J30-50 sand, three specimens were prepared with different initial void ratios of 0.85, 0.75 and 0.65. For F100 sand, the prepared initial void ratio was 0.85.

The volumetric water contents estimated by the TDR system were almost identical to those determined by the burette system, meaning that the volumetric water content could be directly evaluated by the automatic system with the TDR. The use of a membrane instead of a ceramic disk significantly reduced the SWCC test time because of the higher hydraulic conductivity and thin thickness of the membrane. Therefore, multiple-cycles of the SWCC tests can be conducted to characterize the cyclic effects in the SWCC within a relatively short period of experimental time. The multiple-cycles of the SWCC test results showed that the largest hysteresis in the SWCC was observed in the first cycle. The shape and size of SWCC during multiple-cycles after 1.5 cycles (first drying–first wetting–second drying) were almost identical. Thus, at least 1.5 cycles of the SWCC are required for the complete estimation of the soil-water characteristic curve in unsaturated sands. This study demonstrated that the innovative VPPE incorporated with the membrane and TDR system may be an effective method for the complete characterization of multiple-cycles of SWCC in unsaturated geo-materials.

## Figures and Tables

**Table 1:** Index properties of the specimens.

Property	J30-50 Sand	F100 Sand
G_s_^1^	2.62	2.65
e_max_^2^	0.99	0.96
e_min_^3^	0.62	0.59
D_50_^4^	0.46 mm	0.13 mm
C_u_^5^	1.56	1.75
C_c_^6^	1.05	0.89
Soil type	SP ^7^	SP ^7^

^1^ Specific gravity of the soils; ^2^ Maximum void ratio of the specimens; ^3^ Minimum void ratio of the specimens; ^4^ Mean diameter of the specimens; ^5^ Coefficient of uniformity of the specimens; ^6^ Coefficient of curvature on the grain-size distribution curves of the specimens; ^7^ Poorly graded sandy soils classified by the unified soil classification system.

**Table 2:** Specifications of the membrane and ceramic disk.

Classification	Membrane	Ceramic Disk
Pore size	0.2 μm	0.5 μm
Thickness	145 μm	7 mm
Air entry value	350 kPa	500 kPa
Hydraulic conductivity	5.19 × 10^−9^ m/s	7.95 × 10^−10^ m/s
Material	polyethersulfone	ceramic

**Table 3:** Air entry values according to the initial void ratios.

Sand	Initial Void Ratio	Air Entry Value		θ_v_ in Air Entry Value	
		1st Cycle	2nd–5th Cycle	1st Cycle	2nd–5th Cycle
J30-50	0.85	1.0 kPa	0.9 kPa	0.431	0.374
	0.75	1.2 kPa	1.1 kPa	0.399	0.347
	0.65	1.5 kPa	1.4 kPa	0.370	0.321
F100	0.85	5.0 kPa	5.0 kPa	0.412	0.358

**Table 4:** Differences of the volumetric water content according to the initial void ratios.

Sand	Initial Void Ratio	Difference in Volumetric Water Content	
		1st Cycle	2nd–5th Cycle
J30-50	0.85	0.386	0.330
	0.75	0.330	0.270
	0.65	0.279	0.230
